# Do the Prostate-Specific Antigen (PSA) Tests That Are Ordered in Clinical Practice Adhere to the Pertinent Guidelines?

**DOI:** 10.3390/jcm10122650

**Published:** 2021-06-16

**Authors:** Mari Carmen Bernal-Soriano, Lucy Anne Parker, Maite López-Garrigós, Ildefonso Hernández-Aguado, Luis Gómez-Pérez, Juan-Pablo Caballero-Romeu, María Pastor-Valero, Nuria García, Rocío Alfayate-Guerra, Blanca Lumbreras

**Affiliations:** 1 Department of Public Health, University Miguel Hernández de Elche, 03550 Alicante, Spain; lparker@umh.es (L.A.P.); ihernandez@umh.es (I.H.-A.); mpastor@umh.es (M.P.-V.); blumbreras@umh.es (B.L.); 2 CIBER de Epidemiología y Salud Pública (CIBERESP), 28029 Madrid, Spain; lopez_marter@gva.es; 3 Clinical Laboratory Department, University Hospital of San Juan de Alicante, Sant Joan d’Alacant, 03550 Alicante, Spain; 4 Urology Department, University Hospital of San Juan de Alicante, 03550 Alicante, Spain; l.gomez@umh.es; 5 Pathology and Surgery Department, Miguel Hernández University of Elche, 03550 Alicante, Spain; 6 Department of Urology, University General Hospital of Alicante, 03010 Alicante, Spain; caballero_jua@gva.es (J.-P.C.-R.); ngkarb@gmail.com (N.G.); 7 Alicante Institute for Health and Biomedical Research (ISABIAL), 03010 Alicante, Spain; 8 Clinical Laboratory Department, University General Hospital of Alicante, 03010 Alicante, Spain; alfayate_roc@gva.es

**Keywords:** prostate-specific antigen, clinical practice guidelines, prostate cancer, screening

## Abstract

Scientific societies have provided guidelines to reduce PSA-specific harms. We studied the potential non-compliance of PSA testing with current guidelines in general practice. A cross-sectional study of a random sample of 1291 patients with a PSA test was performed between January and April 2018 in primary health care. Patients with a previous prostate cancer diagnosis or those who were being followed-up for previous high PSA values were excluded. Two independent researchers classified whether each test was potentially non-compliant with recommendations. We estimated frequencies of potentially non-compliant PSA determinations and calculated prevalence ratios (PR) to assess their relationship with possible explanatory variables. A total of 66% (95% CI: 62–69%) of PSA requests in asymptomatic patients were potentially non-compliant with the current guideline. This was associated with having a previous diagnosis of neoplasm (PR adjusted by age and life expectancy: 1.18; 95% CI: 1.02–1.37) as well as being a current consumer of tobacco, alcohol, or other drugs (PR: 0.80; 95% CI: 0.67–0.97). Real world data shows that patients are still frequently exposed to overdiagnosis risk with a PSA potentially non-compliant with recommendations. Patients diagnosed with another neoplasm or non-consumers of toxic substances were more exposed, probably due to increased contact with doctors or health-seeking behaviour.

## 1. Introduction

Prostate cancer (PCa) screening using prostate-specific antigen (PSA) has led to a reduction in advanced disease and specific mortality [1,2]. However, it is also associated with overdiagnosis, where we detect true cases of PCa that would not have caused clinical consequences during a man’s lifetime if left untreated [3]. These consequences are significant. Overdiagnosis has been described as ranging from 16% to 50% and increases with increasing age [3], and up to 75–80% of the patients with a positive PSA result do not have cancer (false-positive results) [4]. In addition, biopsies can cause infections and important complications affecting quality of life, such as urinary incontinence and sexual disfunction, or in some cases even sepsis or death [5].

Weighing PSA benefits and harms, the European Association of Urology (EAU) [6] recommended in 2017 that doctors offer an individualized, early-detection strategy to informed patients with good performance status and life expectancy of at least 10–15 years if they have elevated risk of PCa (men > 50 years or 45 years if they are African American or have a PCa family history).

However, recommendations on PSA testing are not always followed. A study analyzed the PSA testing between 2012 and 2017 and showed a frequency of low-value PSA testing ranging from 23.4 to 56.8%, depending upon the specific guideline [7]. The intervals between testing were often shorter than recommended, and screening among younger men without risk factors was frequent [7]. In addition, a different PSA testing behaviour has been described among patients with certain characteristics, such as men with diabetes [8,9], patients with cancer [10], or non-smokers [10,11]. 

The updates of the recommendations by EAU have not been followed by research on the trends and features of PSA testing. In Spain, for example, the Spanish Association of Urology adopts the EAU recommendations and the Spanish Society of Primary Care Physicians recommends PCa screening only for patients who request it and for those with low urinary tract symptoms (LUTS) [12]. Moreover, doctors have to manage a situation where many healthy men want a PSA test because they believe that the test saves their lives [13]. 

Overdiagnosis could be a relevant problem, and it should be noted that PCa incidence rose in Spain from 1990 onwards, mainly due to opportunistic screening, but PCa mortality was only observed to decline slowly from 1998 [14]. Furthermore, we have shown in our environment that PSA testing increased for all age groups but mainly in men younger than 50 years old between 2002 and 2009 [15]. The aim of this study was to examine the potential non-compliance of PSA testing with the current available recommendations in men with and without urinary symptoms attending general practice.

## 2. Materials and Methods

### 2.1. Study Design

We carried out a cross-sectional study to evaluate the adequacy of the PSA tests in primary care of two health areas of Alicante, Spain.

### 2.2. Study Population

The target population were men residing in the catchment area of the two participant hospitals: General University Hospital of Sant Joan d’Alacant (population of 234,424) and General University Hospital of Alicante (population of 255,439). These are referral hospitals for all individuals living in their catchment areas and belongs to the National Health Care System (the majority of the population in Spain uses the National Health System (NHS) as the main medical service (the public health service covers 98.5% of the Spanish population)). PSA determinations are performed centrally in the laboratories of these two hospitals, but the participants come from primary care centres, where their general practitioner requested a PSA test as a routine check-up. We included men over 18 with a PSA determination requested in any primary care consultation from January to April 2018. Patients who were being followed up with for previously high PSA values [16] or who had previously been diagnosed with PCa were excluded. All the PSA tests carried out in these centres are included in the laboratory information system. 

Frequency of PSA Testing in Study Population in 2018

We obtained the number of patients with at least one PSA test during the year 2018 from the laboratory information system. We then calculated overall and age-group-stratified frequency with respect to the male population attached to the health areas included in our study.

### 2.3. Study Size

We estimated a total of 1291 determinations from the two centres to be included in the study according to a previous pilot study with 360 patients in which 70% of the requested PSA determinations were considered to comply with current guidelines with a 95% margin of error and 2% accuracy. We randomly selected 1560 PSA determinations performed in primary care in both health departments included in the study during the first quarter of 2018. A total of 269 patients were excluded due to follow-up of elevated PSA (177), previous PCa diagnosis (48), and not belonging in the health departments studied (44).

### 2.4. Data Collection Procedure

We collected the following variables from the medical records for each patient: (1) Demographic characteristics: Health department, age, and country of birth; (2) patient clinical data: comorbidities, toxic habits (assessed as consumer, non-consumer, or ex-consumer of tobacco, alcohol, and/or drugs), symptoms suggestive of prostate disease, family history of PCa, pharmacological treatment at the moment of the PSA determination, urological tests (echography and rectal exam), prostate surgeries, and the presence of some comorbidities, such as diabetes mellitus or different neoplasm. We also obtained test request information, the number of PSA tests carried out in the last 12 months, and serum PSA concentration (µg/L).

#### 2.4.1. Comorbidity and Life Expectancy Determination 

We obtained the Charlson comorbidity index, which consists of 17 items obtained using the ICD-9MC code system [17], and we used an updated score for each item [18]. We provided the global and categorized score in 4 groups: 0 points, null; 1–2 points, low; 3–4 points, medium; ≥5 points, high. 

We estimated life expectancy (years) by age obtained from the National Institute of Statistics [19]. Finally, each patient was classified as either having a life expectancy of 10 years or more or below 10 years, based on the estimated life expectancy by age in national statistics together with comorbidities and the Charlson comorbidity index.

#### 2.4.2. Definition of a Symptomatic Patient

Patients were classified as symptomatic if they had benign prostatic hypertrophy (BPH) treatments or symptoms suggestive of prostate pathology (hesitancy; weak or interrupted urine flow; frequent urination, especially at night; difficulty emptying the bladder completely; pain or burning when urinating; blood in the urine or semen; persistent pain in the back, hips, or pelvis; pain when ejaculating, and erectile dysfunction) within the 2 years prior to the PSA test. If symptoms were only described at an interval of more than two years prior to inclusion in the study, the patients were considered asymptomatic.

#### 2.4.3. Definition of Potential Non-Compliance of PSA Testing with Current Recommendations

PSA testing in patients classified as symptomatic was considered to comply with guidelines. To ascertain the potential non-compliance of PSA determinations with the current guideline in patients without prostate symptoms, we applied the criteria established by the clinical practice guideline of the EAU [6]. Unfortunately, we were unable to ascertain whether the decision to perform a PSA test was taken jointly by the men and their care providers. For this reason, we applied a conservative definition of PSA non-compliance with the EAU guideline. 

A PSA determination was defined non-compliant if the patient was asymptomatic and presented at least one of the following characteristics:

Under 50 years old (or under 45 years old in men with family history of PCa and/or African American race).

A life expectancy below 10 years.

A previous PSA determination during the last 2 years (±3 months).

#### 2.4.4. Procedure to Assess Potential Non-Compliance

PSA determinations were evaluated separately by two researchers. All the reviewers were trained in the use of the EAU guideline and identification of symptoms suggestive of prostate disease. The reviewers classified each patient as symptomatic or asymptomatic and whether life expectancy was greater than 10 years. Asymptomatic patients were classified as potentially non-compliant with the EAU guideline.

We calculated inter-observer agreement with kappa statistics. The kappa index for pairs of reviewers in the assessment of potential non-compliance of PSA determinations with the European guideline was 0.93 (95% CI: 0.89–0.96). The greatest discordance was obtained in the evaluation of symptoms suggestive of prostatic pathology with a kappa index of 0.54 (95% CI: 0.50–0.59) and 0.68 (95% CI: 0.63–0.73) for current (during preceding 6 months) and previous symptoms, respectively.

In case of disagreement, the case was solved by consensus after review by a third. Persistent disagreements were discussed in a joint meeting with the rest of the team.

### 2.5. Data Analysis Plan

The analysis was performed using the Stata IC 15 (StataCorp LP; College Station, TX, USA). 

We estimated the frequency and 95% confidence interval (95% CI) of the PSA determinations of potentially non-compliant with established recommendations. Characteristics of participants were compared between symptomatic and asymptomatic patient groups using Mann–Whitney test or t-test for continuous variables and the chi-square test for discrete variables. 

We calculated prevalence ratios (PR) to assess the relationship with potential explanatory variables and the magnitude of the association. We used log-Poisson regression to estimate unadjusted and adjusted PR with 95% CI.

## 3. Results

### 3.1. Characteristics of the Study Population

In 2018, 14.5% of men residing in the catchment area of the two participant hospitals presented at least one PSA test. The frequencies of PSA testing in this general population were 1.4%, 7.4%, 34.9%, and 50.7% of men aged: under 45, between 45 and 50, between 51 and 69 and over 70, respectively (data not shown in tables).

According to the sample size estimation, we included 1291 patients with a PSA test performed in primary health care who were not undergoing follow-up of a previously high PSA and did not have a diagnosis of PCa (study population): 537 cases (42%) registered symptoms suggestive of prostate disease and/or were undergoing BPH treatment. Table 1 describes the characteristics of the study population.

**Figure 1:**
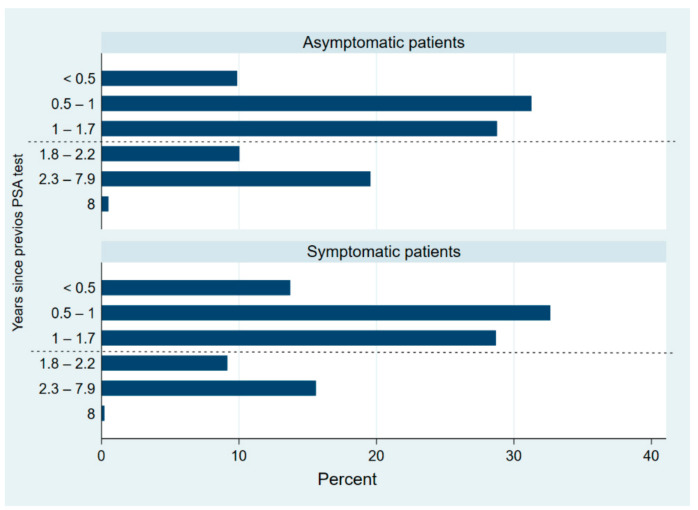
Distribution of interval from previous PSA test by subgroups (asymptomatic and symptomatic patients). Dash line marks minimum recommended interval between PSA tests (approximately two years).

Compared to the asymptomatic men, the symptomatic group presented a significantly higher proportion of patients with a life expectancy under 10 years and/or with a known family history of PCa. A high percentage of patients (779, 60.3%) had a PSA test in a shorter time interval than the available recommendations. This did not appear to be explained by the previous PSA value, as 479 (70%) of patients without a previous PSA value potentially of disease risk (under 1 ng/mL at any age or range from 1 to 2 ng/mL at 60 years of age or more) were retested within two years. Asymptomatic patients (418, 53.7%) were more likely to have had a previous PSA less than two years before compared to symptomatic patients (361, 46.3%), *p* < 0.001 (Figure 1).

### 3.2. Evaluation of the Potential Compliance of PSA Testing with the Recommendations

PSA test was potentially compliant in 61.5% (95% CI: 58.8–64.1%) of participants, of whom 537 (68%) had a PSA test due to the presence of prostate symptoms and/or BPH treatments. A total of 158 (29.4%) symptomatic patients had experienced urinary retention, hesitancy, and/or erectile dysfunction, and 58 (24.7%) of them were undergoing HBP treatment. 

Among asymptomatic patients, 65.9% (95% CI: 62.4–69.2%) had a PSA test that was potentially non-compliant with the European guideline. The previous PSA test time frame was the main cause of non-compliance, with 84.1% (95% CI: 80.6–87.1) of patients having had a previous PSA less than two years before. Nearly one in ten potentially non-compliant PSA test (9.9%; 95% CI: 7.5–12.8) was due to the patient having life expectancy below 10 years.

The age distribution of participants with a PSA determination that was potentially non-compliant with the current guideline was 7.0% (95% CI: 5.1–9.7) under 45, 15.1% (12.2–18.5) between 45 and 50, 53.1 (48.7–57.5) between 51 and 69, and 24.7% (95% CI: 21.1–28.7) were over 70 years old.

In the bivariate analysis (Table 2), asymptomatic patients who were current consumers of tobacco, alcohol, or other drugs showed a lower frequency of potentially non-compliant PSA testing (PR: 0.80 95% CI: 0.67–0.97). Patient characteristics associated with an increased frequency of potentially non-compliant testing were having a high Charlson comorbidity index (PR: 1.57; 95% CI: 1.47–1.68) compared to having a null index or having been diagnosed with another neoplasm (PR:1.37; 95% CI: 1.19–1.58). However, in analysis adjusted by age and life expectancy, the differences detected between those who had been diagnosed with another neoplasm decreased (RP: 1.18; 95% CI: 1.02–1.37), and Charlson comorbidity index was no longer statistically significant. 

Other characteristics associated with an increased frequency of potentially non-compliant testing in adjusted analysis were having a diagnosis of BPH (PR: 1.19; 95% CI: 1.02–1.39) or having a pre-PSA urological test (PR: 1.14; 95% CI: 1.01–1.30) compared to the absence of them. 

## 4. Discussion

Our results showed that potential non-compliance of PSA requests with guideline recommendations was prevalent in clinical practice. In addition, over half of PSA tests were considered to be potentially compliant because of the presence of prostate symptoms and/or BPH treatments. PSA tests may have been carried out in these patients for cancer detection or to assist in treatment and/or decision making processes [20]. According to the National Institute for Health and Care Excellence (NICE), a risk of suspected cancer of 3% or more justifies further examination [21]. Symptoms that have shown a positive predictive value of 3% are urinary retention, hesitancy, and erectile dysfunction [22]. However, these symptoms were not observed in all of the symptomatic patients in our study (29.4% had at least one of these symptoms). The other symptoms recorded may have a lower positive predictive value for PCa, and as such, the PSA petition could be of limited value. 

The symptomatic patients group is of particular interest due to the difficulty in their assessment [23] and the lack of consensus in recommendations as a result of insufficient evidence [24]. Several medical societies recommend PSA testing in patients with LUTS to check for PCa [12,25], although the benefit-to-harm ratio of PSA testing in these patients is unclear [26]. Some findings show a relationship between LUTS and PCa but only for localized cancer [27], while others do not [28,29]. Moreover, symptoms were not associated with PCa-specific mortality [27]. Therefore, symptomatic patients could be exposed to an unnecessary risk of overdiagnosis [24,26]. Other authors recommend considering other cancer risk factors (such as age, family history of PCa, and ethnicity) in PCa screening of patients with LUTS [24]. We found that despite a low proportion of patients reporting a family history of PCa (2%), the majority were in the symptomatic group. However, some symptomatic patients were tested despite having a life expectancy below 10 years, and 37.5% of symptomatic patients were under 45 years old. This would indicate the need for consensus, because most guidelines do not provide clear specifications [12,19,24] for this group. This could be because most of the available evidence on effectiveness of PSA-based screening corresponds to asymptomatic individuals [3].

Age is a key factor to recommend PCa screening. In our study, in contrast to data from 2009 [15], the proportion of patients over 70 (29%) was higher than the proportion under 50 (14%). However, a substantial proportion of potentially non-compliant PSA requests (22%) was found in patients younger than 50 years old without known PCa risk factors despite PCa being a rare neoplasm in men under 50 and 90% of cases occurring in men over 65 [30]. In addition, there is evidence to suggest that the benefit of PCa screening in men over 70 is outweighed by possible associated harms, due to increased risk from false positive results, overdiagnosis, and complications of diagnosis and treatment [3,31]. We found a relevant proportion of testing in men over 70 among patients with LUTS (42%). Older men are more likely to have symptoms [32] and, therefore, to be exposed to a PSA test, which could lead to the detection of tumour without clinical significance. Similarly, different guidelines advocate considering life expectancy when making PSA-based screening decisions [6,33], because men with a life expectancy under 10 years are unlikely to benefit from them given the generally indolent course of the disease. We observed that 11% of men tested had a life expectancy under 10 years, and this proportion was significantly higher in symptomatic patients. These results again reveal a lack of consensus in the recommendations for this group. 

Another measure recommended to reduce harms is to screen men with low PSA levels less frequently [34,35]. Screening biennially with longer inter-screen intervals for men with low PSA levels reduces false-positive tests by 50% [35]. Despite this, we detected a worryingly high proportion of short intervals between tests even in those with a low previous PSA value. Although some authors report that repeating the measurement of PSA in symptomatic men can avoid unnecessary prostatic biopsy [16], they are referring to patients with a high PSA level, and these patients were excluded from our study. 

Furthermore, the latest update of the EAU guideline in 2021 includes the recommendation to offer PSA testing to men carrying BRCA2 mutations from 40 years, but this recommendation was not in force at the time of the study. BRCA2 mutation is a predictor of metastases and worse PCa-specific survival, and the implementation of this test in clinical practice could reduce the overdiagnosis associated with PSA testing. 

We also evaluated potential variables associated with a PSA request that was potentially non-compliant with the current guideline. We speculated that some specific patient characteristics (diabetes diagnosis [8,9], consuming tobacco, alcohol, or other drugs [10,11]) could be associated with lower probability of having a potentially non-compliant PSA test due to lower testing frequency. Nevertheless, although we found no difference in PSA test compliance with the guideline in patients with diabetes diagnoses, those who were current consumers of tobacco, alcohol, and/or other drugs showed 20% lower potential non-compliance. We hypothesized that patients who do not consume tobacco, alcohol, or other drugs take a greater interest in their health, which could lead to greater demand for clinical tests and having a potentially non-compliant PCa screening test. These health-seeking behaviours have been previously associated with a greater use of preventive services [36]. Similarly, we would also have expected those in colorectal cancer screening to be associated with a potentially non-compliant test. However, there was no association, maybe because this screening is an established program, and patients are invited to participate at the same age when PSA screening is considered adequate. Finally, our results showed a direct association between PSA tests that are potentially non-compliant with recommendations and having been previously diagnosed with a different neoplasm. PSA non-compliance in these patients was partially explained by shorter life expectancy, but patients diagnosed with another neoplasm had an 18% higher frequency of potentially non-compliant PSA petitions after adjusting for age and life expectancy. Available evidence has reported that patients with a previous cancer diagnosis were more likely to have a PSA test [10], which could be due to increased contact with healthcare services.

Our research could contribute to an improved use of PSA screening by characterizing its current state in clinical practice. Here, we evaluate the proportion of PSA tests that were ordered that might not comply with the current guidelines. Another important issue would be to consider the patients for whom a PSA test would be recommended but is not ordered. Analysing this issue was beyond the remit of the current study. Furthermore, this study is not without limitations. We retrieved the data from medical files, so its quality is highly dependent on the quality of the information recorded in the files. Fortunately, a recent update to an electronic system in the participating hospitals made it possible for us to access data from different sources (primary, specialized, and hospital care), and this probably improved data completeness and quality of the study. Throughout this manuscript, we refer to potential non-compliance because we could not assess whether shared decision making was used, because medical files do not collect this information. Although guidelines recommend that informed patients make a decision on PSA testing jointly with their clinician, we considered a test to be potentially compliant as long as sociodemographic criteria and frequency intervals were respected. For this reason, the true frequency of non-compliant tests is likely to be underestimated. Moreover, we used a conservative definition of the acceptable interval between tests (two years), even though for low-risk individuals with previously a low PSA test, the recommended interval for retesting is much longer. This too is likely to lead to an underestimation of non-compliance. As the assessment of potential non-compliance may be subject to the subjectivity of the researcher, we decided to perform an observational concordance study. 

We were also unable to establish the aim of PSA testing in the symptomatic group. Almost half of the patients in this group were undergoing BPH treatment, and nine percent of symptomatic patients were asked about their family history of PCa. Therefore, we can assume that these latter were being screened for this condition, but we were not able to know the reason for the PSA test in those with BPH treatment. Another limitation was a potential contamination of the asymptomatic group. Sometimes the BPH diagnosis code in medical records refers to urinary symptoms, and 8% of asymptomatic patients had a BPH diagnosis. This could explain the association of having a potentially non-compliant PSA test in asymptomatic patients with a diagnosis of BPH, prostate surgery, and/or previous urological tests, because patients with these characteristics might have a PSA due to the presence of symptoms. Moreover, some men may have had mild urinary tract symptoms that were not recorded in the clinical files and could be classed in our study as non-symptomatic, but if this was the reason for ordering the PSA test, we would expect the symptomatology to be noted in the files.

Finally, the EAU recommends using the Geriatric-8 and mini-COG tools for health-status screening [37]. However, we did not have all the information necessary to apply them, and instead we used life expectancy together with the Charlson comorbidity index. 

In this study we did not consider interventions carried out after patients had the PSA test. However, as a continuation of this work, a follow-up of the therapeutic process followed by these patients after the PSA test will be carried out to check their compliance with the available recommendations, considering socio-demographic and clinical aspects of the patients.

## 5. Conclusions

Non-compliance with recommendations regarding PCa screening was prevalent. It often takes the form of testing patients more frequently than recommended. The prevalence of a non-compliant test was significantly higher among patients with a previous diagnosis of neoplasm and among non-consumers of tobacco, alcohol, and/or other drugs. In the first group, this is probably due to a higher interaction with medical services, and the second group was characterized by health-seeking behaviour, which led to a greater demand for preventive services. Finally, patients with LUTS showed characteristics with elevated risk of overdiagnosis, although the relationship of LUTS to PCa is controversial. Therefore, recommendations regarding the use of PSA testing in patients with LUTS need to be clarified. Moreover, high non-compliance has been observed in men over 70 years of age, which is also the age group most frequently tested for PSA and has a high prevalence of LUTS. The reason for high non-compliance in these patients could be due to increased interaction with health services, caused by the presence of LUTS or by other comorbidities.

## Figures and Tables

**Table 1:** Description of the study population.

		Symptomatic Patients ^1^ (*n* = 537)	Asymptomatic Patients (*n* = 754)	*p*-Value	Total (*n* = 1291)
Country of birth	Spain (*n*, %)	485 (42.6)	653 (57.4)	0.21	1138 (88.6)
	European (*n*, %)	21 (36.8)	36 (63.2)		57 (4.4)
	Eurasian/Asian (*n*, %)	3 (21.4)	11 (78.6)		14 (1.1)
	African (*n*, %)	10 (38.5)	16 (61.5)		26 (2.0)
	South American (*n*, %)	15 (30.6)	34 (69.4)		49 (3.8)
Age	Mean (SD)	66.0 (12.1)	60.7 (10.7)	<0.001	62.9 (11.6)
	<45 (*n*, %)	21 (37.5)	35 (62.5)	<0.001	56 (4.3)
	45–50 (*n*, %)	39 (30.2)	90 (69.8)		129 (10.0)
	51–69 (*n*, %)	252 (34.8)	473 (65.2)		725 (56.2)
	≥70 (*n*, %)	225 (59.1)	156 (40.9)		381 (29.5)
Life expectancy	<10 (*n*, %)	94 (65.7)	49 (34.3)	<0.001	143 (11.1)
Charlson comorbidity index	Mean (SD)	0.8 (1.4)	0.6 (1.0)	<0.001	0.7 (1.2)
	Null (*n*, %)	302 (37.9)	494 (62.1)	0.01	796 (61.7)
	Low (*n*, %)	190 (46.8)	216 (53.2)		406 (31.4)
	Medium (n, %)	38 (50.0)	38 (50.0)		76 (5.9)
	High (*n*, %)	7 (53.8)	6 (46.2)		13 (1.0)
Prostate surgery	Yes (*n*, %)	47 (78.3)	13 (21.7)	<0.001	60 (4.7)
	<10 years before (*n*, %)	34 (85.0)	6 (15.0)	0.26	40 (74.1)
Family history of PCa	Yes (*n*, %)	14 (66.7)	7 (33.3)	0.02	21 (1.6)
	No (*n*, %)	36 (49.3)	37 (50.7)		73 (5.7)
	Unknown (*n*, %)	487 (40.7)	710 (59.3)		1197 (92.7)
Diabetes mellitus	Yes (*n*, %)	126 (42.3)	172 (57.7)	0.78	298 (23.1)
In CCS program	Yes (*n*, %)	179 (41.1)	256 (58.8)	0.80	435 (33.7)
Diagnosis of another neoplasm	Yes (*n*, %)	25 (47.1)	28 (52.8)	0.40	53 (4.1)
Serum PSA level (ng/mL)	Median (IQR)	1.28 (0.77–2.29)	1.09 (0.66–1.78)	<0.001	1.14 (0.71–1.96)
	Positive result ^2^ (*n*, %)	29 (55.8)	23 (44.2)	0.03	52 (4.0)
Interval from previous PSA test	<2 years (≤21 months)	361 (46.3)	418 (53.7)	<0.001	779 (60.3)
No. PSA tests in 12 months	1 PSA test (*n*, %)	308 (42.3)	420 (57.7)	0.01	280 (26.0)
	2 PSA tests (*n*, %)	130 (46.4)	150 (53.6)		
	≥3 PSA tests (*n*, %)	41 (60.3)	27 (39.7)		68 (6.32)
Previous PSA value potentially of disease risk ^3^	No	283 (41.7)	396 (58.3)	0.01	679 (62.8)
	Yes	200 (49.7)	202 (50.3)		402 (37.2)
Pre-PSA urological test ^4^	Yes	145 (59.2)	100 (40.8)	<0.001	245 (19.0)
BHP	Yes	284 (81.8.)	63 (18.2)	<0.001	347 (26.9)
Health department	1 (*n*, %)	279 (43.2)	367 (56.8)	0.24	646 (50.04)
	2 (*n*, %)	258 (40.0)	387 (60.0)		645 (49.96)
Tobacco	No	112 (37.8)	184 (62.2)	<0.001	296 (30.4)
	Current smoker	104 (36.1)	184 (63.9)		288 (29.6)
	Ex-smoker	195 (50.1)	194 (49.9)		389 (40.0)
Alcohol	No	114 (39.3)	176 (60.7)	0.50	290 (52.6)
	Yes	96 (43.6)	124 (56.4)		220 (39.9)
	Ex	19 (46.3)	22 (53.7)		41 (7.4)
At least 1 toxic habit (alcohol, tobacco, or other drugs)	No	44 (38.6)	70 (61.4)	0.004	114 (13.4)
	Yes	118 (37.6)	196 (62.4)		314 (37.0)
	Ex	207 (49.2)	214 (50.8)		421 (49.6)

^1^ Patients with lower urinary tract symptoms and/or being treated for benign prostatic hypertrophy, ^2^ Serum total PSA level is over 10 ng/mL or between 4 and 10 ng/mL, and value of free PSA/total PSA fraction is under 25%. ^3^ Yes: previous PSA value 1–2 ng/mL if under 60 or previous PSA value above 2 ng/mL for any age; No: previous PSA value 1 ng/mL or 1 to 2 ng/mL if over 60. ^4^ Echography or rectal exam during last 2 years. Abbreviations: PCa, prostate cancer; CCS, colorectal cancer screening; BPH, benign prostatic hypertrophy; IQR, interquartile range.

**Table 2:** Relationship between prostate-specific antigen request potentially compliant with the EAU guideline and potential explanatory variables in asymptomatic patients.

		PSA Tests Potentially Non-Compliant with the EAU Guideline (*n* = 754)			
		No, *n* (%)	Yes, *n* (%)	Crude PR	Adjusted PR ^1^
Country of birth	Spain	215 (32.9)	438 (67.1)	Ref	Ref
	European	13 (36.1)	23 (63.9)	0.95 (0.74–1.22)	0.95 (0.74–1.22)
	Eurasian or Asian	6 (54.5)	5 (45.5)	0.68 (0.35–1.30)	0.69 (0.35–1.32)
	African	7 (43.7)	9 (56.3)	0.84 (0.54–1.30)	0.85 (0.55–1.33)
	South American	15 (44.1)	19 (55.9)	0.83 (0.61–1.13)	0.85 (0.62–1.15)
Charlson comorbidity index	Null	179 (36.2)	315 (63.8)	Ref	Ref
	Low	68 (31.5)	148 (68.5)	1.07 (0.96–1.20)	1.06 (0.94–1.19)
					1.04 (0.93–1.17) ^2^
	Medium	10 (26.3)	28 (73.7)	1.14 (0.93–1.41)	1.11 (0.90–1.38)
					0.99 (0.80–1.23) ^2^
	High	0	6 (100.0)	**1.57 (1.47–1.68)**	**1.49 (1.34–1.66)**
					1.08 (0.92–1.27) ^2^
PSA value potentially of disease risk ^4^	No	105 (26.5)	291 (73.5)	Ref	Ref
	Yes	53 (26.2)	149 (73.8)	1.00 (0.91–1.11)	1.02 (0.92–1.12)
Diabetes mellitus	No	207 (35.6)	375 (64.4)	Ref	Ref
	Yes	50 (29.1)	122 (70.9)	1.10 (0.98–1.23)	1.08 (0.96–1.21)
BPH	No	246 (35.6)	445 (64.4)	Ref	Ref
	Yes	11 (17.5)	52 (82.5)	**1.28 (1.13–1.45)**	**1.28 (1.13–1.45)**
					**1.19 (1.02–1.39) ^3^**
Prostate surgery	No	255 (34.4)	486 (65.6)	Ref	Ref
	Yes	2 (15.4)	11 (84.6)	**1.29 (1.02–1.64)**	1.22 (0.96–1.56)
					1.10 (0.86–1.41) ^3^
Pre-PSA urological test ^5^	No	234 (35.8)	420 (64.2)	Ref	Ref
	Yes	23 (23.0)	77 (77.0)	**1.20 (1.06–1.35)**	**1.18 (1.04–1.34)**
					**1.14 (1.01–1.30) ^3^**
Undergoing CCS program	No	173 (34.7)	325 (65.3)	Ref	Ref
	Yes	84 (32.8)	172 (67.2)	1.03 (0.93–1.15)	1.02 (0.92–1.14)
Diagnosis of another neoplasm	No	254 (35.0)	472 (65.0)	Ref	Ref
	Yes	3 (10.7)	25 (89.3)	**1.37 (1.19–1.58)**	**1.34 (1.16–1.55)**
					**1.18 (1.02–1.37) ^2^**
At least 1 toxic habit (alcohol, tobacco, or other drugs)	No consumer	19 (27.1)	51 (72.9)	Ref	Ref
	Current consumer	81 (41.3)	115 (58.7)	**0.80 (0.67–0.97)**	**0.81 (0.67–0.98)**
	Ex-consumer	67 (31.3)	147 (68.7)	0.94 (0.80–1.12)	0.93 (0.78–1.10)
Health department	1	128 (34.9)	239 (65.1)	Ref	Ref
	2	129 (33.3)	258 (66.7)	1.02 (0.92–1.13)	1.02 (0.92–1.13)
Total		257 (34.1)	497 (65.9)		

Statistically significant values in bold. ^1^ Adjusted by age. ^2^ Additionally adjusted by life expectancy. ^3^ Additionally adjusted: model included BPH, prostate surgery, pre-PSA urological test, and age. ^4^ Yes: previous PSA value 1–2 ng/mL if under 60 or previous PSA value above 2 ng/mL for any age; No: previous PSA value 1 ng/mL or 1 to 2 ng/mL if over 60. ^5^ Echography or rectal exam during last 2 years. Abbreviations: EAU, The European Association of Urology; PR, prevalence rate; BPH, benign prostatic hypertrophy; CCS, colorectal cancer screening.

## Data Availability

The data presented in this study are available on request from the corresponding author.
